# The Impact of Resistance Exercise on Muscle Mass in Glioblastoma in Survivors (RESIST): Protocol for a Randomized Controlled Trial

**DOI:** 10.2196/37709

**Published:** 2022-05-04

**Authors:** Melanie R Keats, Scott A Grandy, Christopher Blanchard, Jonathon R Fowles, Heather F Neyedli, Adrienne C Weeks, Mary V MacNeil

**Affiliations:** 1 Division of Kinesiology School of Health and Human Performance Dalhousie University Halifax, NS Canada; 2 Division of Medical Oncology Department of Medicine Dalhousie University Halifax, NS Canada; 3 Beatrice Hunter Cancer Research Institute Halifax, NS Canada; 4 Department of Medicine Faculty of Medicine Dalhousie University Halifax, NS Canada; 5 School of Kinesiology Acadia University Wolfville, NS Canada; 6 Department of Psychology and Neuroscience Dalhousie University Halifax, NS Canada; 7 Division of Neurosurgery Department of Surgery Dalhousie University Halifax, NS Canada

**Keywords:** glioblastoma, myopathy, resistance exercise, functional fitness, quality of life, intervention, randomized controlled trial

## Abstract

**Background:**

Glioblastoma is the most common primary brain malignancy in adults, accounting for approximately 48% of all brain tumors. Standard treatment includes radiation and temozolomide chemotherapy. Glioblastomas are highly vascular and can cause vasogenic brain edema and mass effect, which can worsen the neurologic symptoms associated with the disease. The steroid dexamethasone (DEX) is the treatment of choice to reduce vasogenic edema and intracranial pressure associated with glioblastoma. However high-dose DEX or long-term use can result in muscle myopathy in 10%-60% of glioblastoma patients, significantly reducing functional fitness and quality of life (QOL). There is a wealth of evidence to support the use of exercise as an adjuvant therapy to improve functional ability as well as help manage treatment-related symptoms. Specifically, resistance training has been shown to increase muscle mass, strength, and functional fitness in aging adults and several cancer populations. Although studies are limited, research has shown that exercise is safe and feasible in glioblastoma populations. However, it is not clear whether resistance training can be successfully used in glioblastoma to prevent or mitigate steroid-induced muscle myopathy and associated loss of function.

**Objective:**

The primary purpose of this study is to establish whether an individualized circuit-based program will reduce steroid-induced muscle myopathy, as indicated by maintained or improved functional fitness for patients on active treatment and receiving steroids.

**Methods:**

This is a 2-armed, randomized controlled trial with repeated measures. We will recruit 38 adult (≥18 years) patients diagnosed with either primary or secondary glioblastoma who are scheduled to receive standard radiation and concurrent and adjuvant temozolomide chemotherapy postsurgical debulking and received any dose of DEX through the neurooncology clinic and the Nova Scotia Health Cancer Center. Patients will be randomly allocated to a standard of care waitlist control group or standard of care + circuit-based resistance training exercise group. The exercise group will receive a 12-week individualized, group and home-based exercise program. The control group will be advised to maintain an active lifestyle. The primary outcome, muscle myopathy (functional fitness), will be assessed using the Short Physical Performance Battery and hand grip strength. Secondary outcome measures will include body composition, cardiorespiratory fitness, physical activity, QOL, fatigue, and cognitive function. All measures will be assessed pre- and postintervention. Participant accrual, exercise adherence, and safety will be assessed throughout the study.

**Results:**

This study has been funded by the Canadian Cancer Society Atlantic Cancer Research Grant and the J.D. Irving Limited–Excellence in Cancer Research Fund (grant number 707182). The protocol was approved by the Nova Scotia Health and Acadia University’s Research Ethics Boards. Enrollment is anticipated to begin in March 2022.

**Conclusions:**

This study will inform how individualized circuit-based resistance training may improve functional independence and overall QOL of glioblastoma patients.

**Trial Registration:**

ClinicalTrails.gov NCT05116137; https://www.clinicaltrials.gov/ct2/show/NCT05116137

**International Registered Report Identifier (IRRID):**

DERR1-10.2196/37709

## Introduction

### Background

Glioblastoma is the most common primary malignant brain tumor in adults [1]. With a median age at diagnosis of 64 years and an incidence rate of approximately 4 per 100,000 people in Canada [2] (4.53/100,000 in Atlantic Canada), glioblastoma is an aggressive tumor with an unfavorable prognosis, making it a significant public health issue [3]. Despite advances in surgical, radiotherapy, and chemotherapeutic approaches, the median survival remains discouraging, ranging from 14 months to 22 months [4].

Glioblastoma, a highly vascular tumor, is known to cause vasogenic brain edema and mass effect, which can worsen underlying neurologic deficits [5,6]. The administration of steroids, most commonly dexamethasone (DEX), is a well-established standard of care in the treatment of vasogenic edema and increased intracranial pressure associated with central nervous system tumors [7]. Steroids are often started in the preoperative setting and continued for varying lengths of time depending on the degree of surgical debulking and response to subsequent treatment. In clinical trials, between 40% and 70% of patients require steroids at the start of radiochemotherapy [8,9]. Although DEX has become the mainstay in the management of tumor-related edema, high doses and long-term use can result in a multitude of harmful side effects, including but not limited to myopathy (muscle weakness) [10,11].

About 10% of glioblastoma patients will develop clinically significant steroid-induced myopathy after just 2 weeks of high-dose therapy [12]. With long-term steroid use, as many as 50% to 60% of patients will suffer from myopathy [13]. This risk is further compounded by the age of the patient, as older adults are at higher risk of myopathy given their lower baseline muscle mass [10,13,14]. In conjunction with fatigue and inactivity [13,14], steroid-induced myopathy results in progressive muscle weakness and functional decline. This can cause balance problems, increase the risk of falls and fractures, and make it more difficult to rise from a chair and climb stairs. These changes can result in a loss of independence and a reduced quality of life (QOL) [7,14].

In addition to the neurologic and functional deficits caused by the tumor, patients experience an array of complex symptoms including cognitive difficulties, sleep disturbance, seizures, emotional distress, and fatigue [15]. Tumor location, treatment, and supportive medications all contribute to these troubling symptoms, which affect QOL. Although glioblastoma prognosis has been associated with numerous clinical factors (eg, patient age, extent of maximal safe resection), functional impairment has been consistently shown to be a significant predictor of greater symptom burden [16,17] and survival [4,14,18,19]. As an incurable disease, it is important to consider factors such as symptom management, maintaining functional independence, and preserving or improving QOL [14,20]. Of note, the ability to walk and perform physical tasks was noted as one of 3 top priorities for brain tumor patients [7].

### Exercise and Cancer

A growing body of evidence demonstrates that exercise is associated with improved symptom management, physical and mental well-being, QOL, and survival [21-23]. As with any exercise intervention, adverse events can and do occur; however, safety studies have demonstrated that tailored exercise interventions (adapted to disease, treatment, and patient characteristics) are safe both during and post cancer treatment [21,22] The strength of the evidence has led to the development of cancer-specific exercise guidelines advising that all cancer patients should avoid inactivity [23,24]. Notwithstanding, the bulk of the existing literature is limited to the most common cancers and in patients with early-stage disease [23].

Glioblastoma patients are a heterogenous group with complex health care needs, and exercise interventions are often assumed unfeasible or even contraindicated. As such, glioblastoma patients are underrepresented in the exercise oncology literature [25]. Notwithstanding, animal studies have shown that exercise in mice with glioblastoma can delay motor dysfunction. In humans with brain cancer, preliminary data suggest that exercise is safe, feasible, and likely beneficial. Specifically, improvements in symptom severity, body composition, activity levels, aerobic capacity, neurocognitive functioning, headaches, mental health, and QOL have been reported [26,27]. Clinically meaningful changes have also been reported for lower body strength, balance, fatigue, and sleep following exercise [26,27]. A study by Ruden and colleagues [28] found that exercise in adults with recurrent glioma was an independent predictor of survival.

### Resistance Exercise

Even with the high incidence of steroid-induced myopathy in glioblastoma patients, few studies have explored the impact of resistance-based exercise on functional status and patient-reported outcomes in this population. Notwithstanding, there is a growing body of evidence that resistance training can mitigate or prevent progressive loss of muscle mass and strength (ie, steroid-induced myopathy or secondary sarcopenia). In this proposal, sarcopenia and myopathy are used interchangeably. Sarcopenia denotes a syndrome characterized by a progressive loss of muscle mass and strength, leading to physical dysfunction, reduced QOL, and risk of death. For glioblastoma patients, sarcopenia can be attributed to both high and prolonged steroid use, reduced activity due to fatigue, deconditioning and functional loss, and advancing age [10,13,14].

Despite the clinical importance of sarcopenia, the management of this disease remains challenging [29]. In general, resistance training is considered a first-line treatment to manage sarcopenia [29,30]. Progressive resistance training using low-to-moderate intensity, weight-bearing exercises has been shown to improve muscle mass, strength, and functional capacity [31-33]. Resistance training has also been shown to improve gait speed and balance and reduce the risk of falls in the elderly [34]. Similarly, resistance training has been demonstrated to be an important element in reversing sarcopenia in cancer patients. For example, resistance training has been shown to reduce body fat and improve muscle mass, strength, functional ability, and QOL in prostate cancer survivors [35-37]. Similarly, resistance training in breast cancer survivors receiving adjuvant chemotherapy has been shown to reverse sarcopenia [38]. Although resistance training has been suggested to be the most important element in managing sarcopenia, others have noted that the additive effects of a combined aerobic and resistance training exercise lead to the greatest improvements in physical functioning [39,40].

We found 3 studies that explored the role of exercise in brain tumor patients; 2 with stable grade II-III gliomas demonstrated that a home-based, remotely supervised exercise program can improve cardiorespiratory fitness and cognitive function [41,42]. Capozzi et al [43] reported that a once-a-week supervised session of combined aerobic and resistance training exercise improved functional performance in grade I-III brain tumor patients. Despite the well-established benefits of both aerobic exercise and resistance training, there remains a hesitancy to prescribe any form of exercise in certain cancer populations, including those with glioblastoma. The concern regarding exercise prescription for glioblastoma is likely the result of the neurological deficits associated with the cancer as well as the lack of clinical trials examining the benefit of exercise in this population [44]. Although safety and feasibility of resistance training have not been widely studied in a glioblastoma population, 3 case studies found that resistance training is safe and feasible for those with glioblastoma [45-47]. Most recently, Halkett et al [48] piloted a tailored exercise intervention for glioblastoma patients scheduled to receive radiation and chemotherapy. Patients participated in a 1-hour combined aerobic-resistance training sessions 3 times a week over the course of 7 weeks of chemotherapy. Sessions were supervised by an exercise physiologist and delivered at the treating hospital. At the conclusion of the trial, patients identified both challenges (ie, managing symptoms, juggling exercise and treatment, difficulties engaging with the program) and benefits (ie, personalized program, improvements in physical and psychological health, regaining a sense of control, interacting with people) associated with participation [48]. In brief, these studies show that glioblastoma patients are willing and able to safely engage in supervised exercise and, in doing so, show improvements in functional performance and QOL. Although promising, these studies are limited to small sample sizes (n=1-19) and nonrandomized designs. The primary purpose of this study will be to examine the impact of a tailored, circuit-based resistance training program on functional fitness for glioblastoma patients on active treatment. Secondary outcomes will include safety, exercise adherence, body composition, cardiopulmonary function, activity levels, general health, fatigue, cognitive functioning, and QOL.

## Methods

### Ethics Approval and Trial Registration

Ethics approval has been obtained from the research ethics boards at Nova Scotia Health (ROMEO REB File number 1027521) and Acadia University (REB File number 22-03).

### Study Design and Procedures

**Figure 1:**
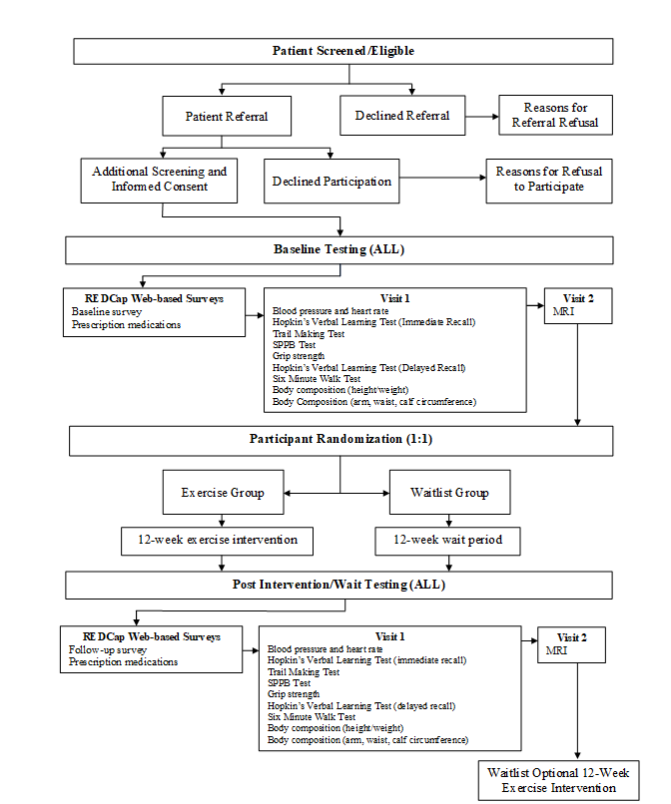
Participant flow.

This manuscript has been written in accordance with the Standard Protocol Items for Randomized Trials (SPIRIT) 2013 guidelines [49]. This is a 2-armed, randomized controlled trial with repeated measures. Eligible participants will be referred to our exercise lab located at the Nova Scotia Cancer Center, Halifax, Nova Scotia, Canada. Here, they will provide informed consent, complete a baseline survey, and undergo a comprehensive functional fitness assessment conducted by a clinical exercise physiologist (CEP). Following baseline assessments, participants will be randomized 1:1 to either the 12-week exercise intervention (EX) or standard of care, waitlist control group (CON). Block randomization in groups of 4 will be used to ensure an equal balance of participants in each group throughout the study period. The allocation sequence will be concealed from the project coordinator who will be involved in assigning patients to groups. Given the nature of the exercise-based intervention, it not possible to blind participants or the CEPs to group allocation. All participants will return to the exercise lab at the end of the 12-week study to complete a final assessment (Figure 1).

### Participants

We will seek to recruit 38 adult (≥18 years) patients diagnosed with either primary or secondary glioblastoma who are scheduled to receive standard radiation (>65 years old, 3 weeks; <65 years old, 6 weeks) and concurrent and adjuvant temozolomide chemotherapy postsurgical debulking or biopsy. Patients will be recruited through the neurooncology clinic at the Nova Scotia Health Cancer Center. Participants will be screened by MVM and deemed eligible based on the following criteria: (1) histologically confirmed diagnosis of either primary or secondary glioblastoma, (2) received any dose of DEX, (3) Karnofsky Performance Status (KPS) >70, (4) English fluency, (5) physician approval, and (6) willingness to travel to Halifax to participate. Exclusion criteria include (1) unstable or symptomatic cardiac or pulmonary disease, injury, or comorbid disease that precludes ability to safely exercise; (2) significant cognitive limitations or lacking sufficient mental capacity to give consent (eg, mental health conditions such as schizophrenia, dementia, and brain injury); and (3) uncontrolled seizures associated with impaired awareness. Capacity to provide consent will be assessed through a verification of comprehension through several embedded questions in the informed consent. These questions will ascertain a patient’s ability to understand what is being asked of them as a potential research participant.

### Standard of Care

Standard treatment for glioblastoma after biopsy or surgical debulking involves a combination of daily oral temozolomide chemotherapy given with daily radiation (5 days/week) for 3 weeks to 6 weeks for a final radiation dose of 40-60 Gy given in 15-30 fractions, respectively. Generally, patients <65 years old receive 6 weeks of radiation with daily temozolomide. Due to poor tolerance of this high dose, patients >65 years old, or those with aggressively growing tumors, receive the same general regime but for 3 weeks. For those between the ages of 65 years and 70 years, 3 weeks or 6 weeks of radiation is chosen based on underlying fitness. Radiation can only be given at the cancer center, so patients attend appointments in person each day. It is during this time that patients may have their steroid dose increased to control the edema associated with radiation. Once radiation is completed, there is a 1-month recovery period, during which there is further adjustment of steroid dose, as needed. Stable patients will continue temozolomide on its own, where it is taken for 5 days a month for 6 months. Throughout this time, patients are examined monthly and monitored every 2 months with magnetic resonance imaging (MRI) of the brain to assess tumor response and guide requirements for further steroid adjustment.

All study patients will receive standard of care treatment. Participants allocated to the CON group will be advised to maintain an active lifestyle but will not receive any formal exercise prescription. CON participants will be given the opportunity to participate in the same intervention following the 12-week control period.

### Intervention and Study Setting

Following baseline assessments, participants allocated to the EX group will meet with a CEP at our hospital-based exercise lab. Here, participants will receive an individually tailored, circuit-based resistance training program. Circuit-based resistance training is a common training method used to foster aerobic fitness, muscular endurance, and strength, as well as neuromuscular adaptations in 1 workout. Circuit-based resistance training is comprised of several sets of different exercises with little rest in between each set [50]. EX participants will be asked to return to the lab for 3 to 4 supervised sessions per week for 12 weeks. Sessions will be designed to coincide with standard of care visits to reduce participant burden.

**Figure 2:**
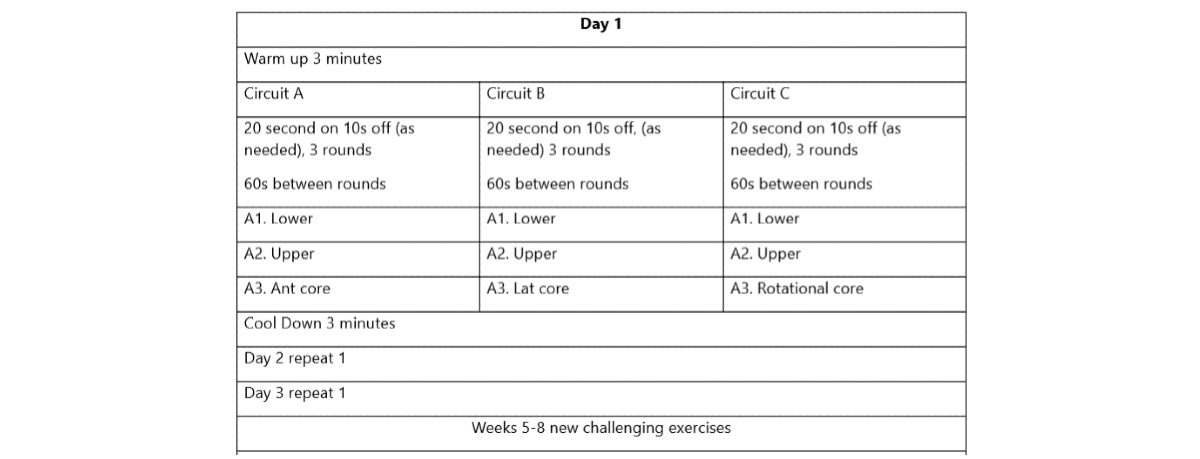
Introductory resistance-based exercise circuit (whole body).

**Figure 3:**
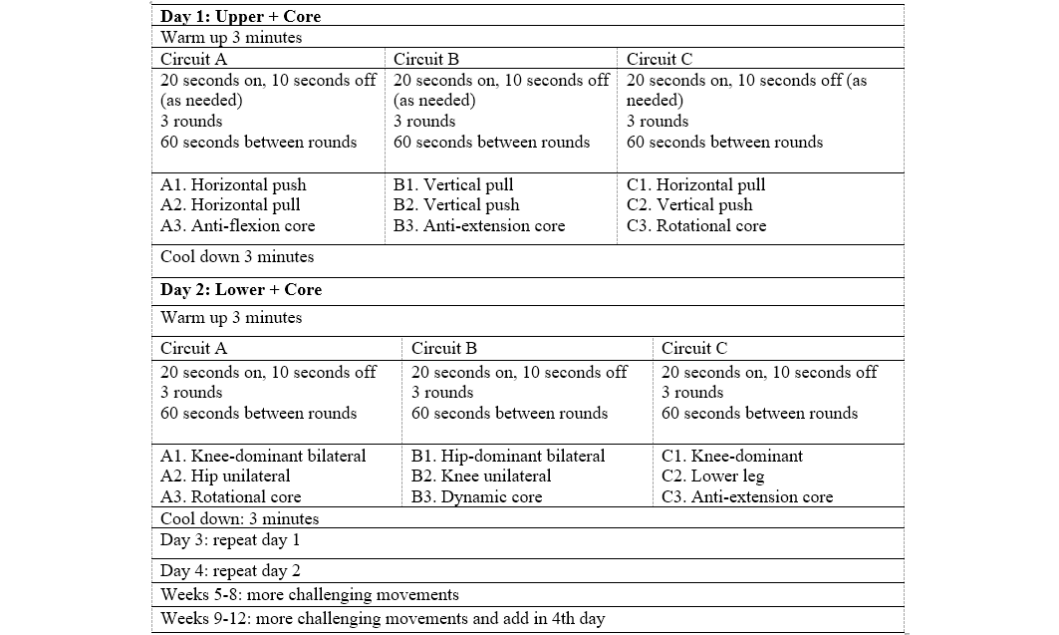
Intermediate and advanced resistance-based exercise circuit.

Participants will begin with a light-to-moderate-intensity (3-6 on the 10-point Borg Scale), systematically progressed, circuit-based resistance training program for 20 minutes to 30 minutes per session. Each session will consist of 3 circuits. Each circuit will involve 3 sets of 3 different exercises (20-second intervals for a total of 1 minute per set; short rest between exercises will be provided as needed). Each set will be followed by a 1-minute break. Figures 2 and 3 describe a sample training program and progression. Following the first 3 weeks to 6 weeks of supervised exercise sessions (coinciding with treatment protocol), participants allocated to the EX group will be asked to attend a minimum of 1 in-person session per week for the remaining 6 weeks to 9 weeks of the 12-week program. To reduce participant burden, participants will be offered the option of completing the remaining 2 to 3 weekly exercise sessions at home (supported virtually as needed or preferred). For those living in the Western zone, following the first 3 weeks to 6 weeks of the program, delivered at our hospital-based exercise lab, in-person programming will also be offered at our partner site in Wolfville, Nova Scotia (Acadia University) under the supervision of a CEP. Participants will have the option of completing more than one or all weekly exercise sessions in person at either site. Initial exercise prescriptions will be developed and modified as needed by a CEP in accordance with the participant’s health and fitness status. Physician referral, individually tailored, and short, lighter intensity resistance training sessions have been designed to foster program adoption and adherence [51].

### Measures

#### Sociodemographic Characteristics

Sociodemographic characteristics will include age, sex, gender, level of education, ethnicity, annual household income, and employment status.

#### Medical History and Performance Status

Medical information will include surgical date and procedure (biopsy/resection), tumor characteristics, treatments received, neurological deficits, treatment-related side effects using the International Common Toxicity Criteria Adverse Event (CTCAE) [52], comorbidities, and prescription medications. Performance status will be evaluated using the KPS [53], assessed by MVM at the time of study enrollment and following the completion of the study.

The primary outcome measure is steroid-induced myopathy as determined by functional performance. Functional performance will be assessed using the Short Physical Performance Battery (SPPB) test [54,55] and handgrip strength [14]. The SPPB uses tasks that mimic activities of daily living and examines 3 areas of lower body function: gait speed, balance, and muscular endurance. Grip strength will be evaluated using a handheld dynamometer. Mean handgrip strength will be calculated from a total of 4 measurements (2 with the right hand and 2 with the left hand) and recorded to the nearest 0.1 kg [56].

Secondary outcome measures will include body composition, aerobic fitness, vital measurements (ie, resting blood pressure and heart rate), physical activity, general health, QOL, fatigue, and cognitive functioning. Participant accrual, attrition, program adherence, reasons for missed sessions, and adverse events will be documented. Body composition measures will include body mass index (kg/m^2^), arm circumference, waist circumference, calf circumference [57,58], and muscle mass and quality. Muscle density and intramuscular adipose tissue will be assessed using whole-body MRI [59]. Flex processing will be used to construct muscle, fat, and water images. All scans and imaging processing will be performed using a 3T MRI and associated software at Biotic Imaging for Life. Aerobic fitness will be measured using the 6-minute walk test (6MWT) [60]. The 6MWT is an effective measure of physical functioning in recurrent primary malignant gliomas [61]. General health will be assessed using the EQ-5D-5L [62,63]. The EQ-5D-5L is a valid, widely used tool that measures patient health across 5 dimensions (mobility, self-care, usual activities, pain/discomfort, and anxiety/depression) [62,63]. QOL will be assessed using the 50-item Functional Assessment of Cancer Therapy-Brain (FACT-Br), which provides scores for physical, functional, emotional, and social and family well-being as well as a brain cancer–specific subscale [64]. Fatigue will be measured using the 13-item Functional Assessment of Chronic Illness Therapy-Fatigue (FACIT-F) [65]. Both the FACT-Br and FACIT-F have been extensively validated and are reliable and widely used tools in the oncology setting [65-67]. Self-reported exercise will be assessed using the Godin Leisure-Time Exercise Questionnaire (GLTEQ) [68-70]. The GLTEQ is a well-validated questionnaire that assesses the frequency and duration of mild, moderate, and vigorous leisure-time exercise participation [70]. Objective physical activity will be captured through a wristworn Garmin activity tracker. Cognitive functioning will be assessed using the Trail Making Test [71] for executive function and the Hopkins Verbal Learning Test-revised for memory [72-74].

### Sample Size

Sample size is based on a meaningful change (effect size 0.30) in physical performance [75]. A sample of 24 (12 per group) will allow us to detect differences as small as 0.3 points to 0.8 points on the SPPB with 80% power based on an ɑ=.05 with no sphericity correction. Although randomization should account for variations in sex, we will strive to enroll 38 participants (see the Feasibility section) to explore this covariate.

### Feasibility

Our medical oncologist (MVM) typically sees 45 to 50 new glioblastoma patients each year, and roughly half of these patients live outside of Halifax. To increase our reach, we have partnered with JRF (Acadia University, Wolfville) to accommodate patients within the Western zone who may not be able or willing to travel to the main Halifax site. Anticipating a 60%-65% accrual rate, a sample of 12 patients to 13 patients per year is deemed feasible. Should participant accrual be more challenging than anticipated, we will open recruitment to grade III gliomas.

### Statistical Analyses

Descriptive statistics will be used to describe the population, accrual, program adherence, and safety. Study outcome measures will be assessed pre- and postintervention and will be analyzed using an intention-to-treat approach. A multiple imputation model will be used to account for any missing data. All participants will then be entered into a mixed effects model with participant group assignment at randomization and time point (pre- and posttest) as fixed factors and participant entered as a random factor. Due to feasibility in recruiting participants in the allotted time, the study will not be fully powered to detect sex-based differences; however, effect sizes associated with the intervention will be calculated and presented for each sex.

## Results

This study brings together a multidisciplinary team with extensive expertise in research and clinical practice within the fields of exercise oncology (MRK, SAG); exercise measurement, evaluation, and prescription (MRK, SAG, JRF); exercise physiology (SAG, JRF); behavioral medicine (MRK, CB); medical oncology (MVM); neurosurgery (ACW); research methods and statistics (HFN, CB); clinical trials (all authors); and knowledge translation (all authors). The study team has previously collaborated on research projects including 2 ongoing exercise trials for cancer survivors (Activating Cancer Communities through an Exercise Strategy for Survivors [76], and EXercise for Cancer to Enhance Living well [77]). This study has been funded by the Canadian Cancer Society Atlantic Cancer Research Grant and the J.D. Irving, Limited-Excellence in Cancer Research Fund (grant number 707182). As of March 16, 2022, 1 patient had been enrolled.

## Discussion

### Overview

Glioblastoma is a devastating diagnosis with a high mortality rate and rapid loss of function and independence; thus, it is imperative to consider factors such as symptom management, maintaining functional independence, and preserving or improving QOL for the duration of the patients’ lives. Physical exercise has been well-established to improve functional capacity and QOL in the cancer population. However, few studies have evaluated the efficacy of exercise in mitigating the debilitating physical and functional deficits experienced by glioblastoma patients. Specifically, given the high symptom burden and lack of clinical trials examining the efficacy of exercise in this population, there remains a hesitancy to prescribe any form of exercise. Although the safety and feasibility of exercise for glioblastoma patients have not been widely studied, emerging evidence suggests that brain cancer patients are interested and able to safely engage in physical exercise. Employing a much-needed randomized design, this study will examine the impact of a tailored, circuit-based resistance training program on functional fitness for glioblastoma patients on active treatment. It is anticipated that this study will demonstrate that resistance training not only is safe and feasible for those with glioblastoma but also significantly improves functional status by protecting against steroid-induced myopathy, thereby helping glioblastoma patients maintain their independence, which could lead to marked improvements in QOL.

### Dissemination Plan

The proposed work is supported by a multidisciplinary team with the expertise, experience, and infrastructure to ensure the successful implementation of the study. Building on over 13 years of exercise programming for cancer patients and survivors in Nova Scotia, and with the support of the Nova Scotia Health Cancer Care Program, we have built a strong provincial outreach and patient engagement strategy that has provided the necessary foundation for province-wide implementation and dissemination.

Our knowledge translation plan will be directed by 2 main objectives: (1) to raise awareness and understanding of the benefits of physical activity or exercise for glioblastoma patients and their families and (2) to advance the field of study. To address our first objective, following the conclusion of the study, we will create and promote a webinar to share lessons learned with glioblastoma patients and their families. We will also summarize the study findings in a lay document that will be posted on our website [78]. To address objective 2, we will publish our findings in peer-reviewed publications and will present our findings at local, national, and international conferences.

### Conclusions

In brief, this study will play a critical role in better understanding how physical exercise can foster an optimal level of function, independence, and QOL in glioblastoma patients.

## Appendix

Multimedia Appendix 1 Grant agency peer review - reviewer 1.

Multimedia Appendix 2 Grant agency peer review - reviewer 2.

Multimedia Appendix 3 Grant agency peer review - reviewer 3.

## Abbreviations

6MWT: 6-minute walk test
CEP: clinical exercise physiologist
CON: waitlist control group
CTCAE: Common Toxicity Criteria Adverse Event
DEX: dexamethasone
EX: exercise group
FACIT-F: Functional Assessment of Chronic Illness Therapy-Fatigue
FACT-Br: Functional Assessment of Cancer Therapy-Brain
GLTEQ: Godin Leisure-Time Exercise Questionnaire
KPS: Karnofsky Performance Status
MRI: magnetic resonance imaging
QOL: quality of life
SPIRIT: Standard Protocol Items for Randomized Trials
SPPB: Short Physical Performance Battery
