# Supplementary material for: The Impact of Resistance Exercise on Muscle Mass in Glioblastoma in Survivors (RESIST): Protocol for a Randomized Controlled Trial
Source: JMIR Res Protoc. 2022 May 4;11(5):e37709. doi: 10.2196/37709 (PMC9118089; doi:10.2196/37709)
Supplement: Multimedia Appendix 1 [file resprot_v11i5e37709_app1.pdf]

Applicant: Keats, Melanie
Institution: Dalhousie University
Application: The impact of Resistance ExerciSe on muscle mass in GIIoblaSToma survivors (RESIST)
Review Team: Health Services/Social, Cultural, Environmental and Population Health
Review Report: Primary Reviewer Report

Brief summary or abstract:

Glioblastoma (GBM) is the most common malignant brain tumor in adults; its clinical sequelae is associated with debilitating physical side effects and treatment toxicities. Little is known on the impact of exercise on GBM patients undergoing active treatment.

A two-armed RCT on 38 adults undergoing active treatment is proposed: 1) circuit-based resistance exercise or 2) standard of care. The intervention is over 12-weeks (with resistance exercise occurring every 3-4 days/week). The primary outcome is functional performance (using the Short Physical Performance Battery and handgrip strength); while secondary outcome measures include body composition, aerobic fitness, physical activity, general health, QoL, fatigue and cognitive function. All outcome measures will be assessed at study enrollment and at 12-week.

Critique (consider research strategy, investigator(s), environment, and relevance):

RESEARCH STRATEGY

Overall, I think the need for a trial was justified. Given the debilitating physical side-effects and treatment toxicities associated with GBM, the benefits of exercise in this patient population have been under-investigated.

Careful thought went into ensuring that the intervention will occur during the same time as treatment which lowers participant burden.

However, I have several concerns:
1. Reference 26-28 was used to support that humans with brain cancer can safely exercise and that exercise may have positive effects on outcomes such as survival, and physical, behavioural and cognitive functioning. However, Reference 26 also highlighted that there are already two ongoing RCT on this topic: NCT03390569 in Toronto (exercise and progression-free survival; plus, many secondary outcome measures including physical function and QoL) and NCT03775369 in Switzerland (physical activity and QoL). The proposed study appears to overlap greatly with the ongoing trial in Toronto. Thus, what is the justification of conducting yet another trial given the results of the ongoing trial will be available in December 2021?
2. Overall, I think pilot data was lacking to support:
a. That the intervention will be tolerable e.g., exercising 3-4 days/week while on treatment
b. That patients will adhere the intervention (i.e., minimize loss-to-follow-up)
c. A 12-week intervention; why 12 weeks? Is it enough time to observe changes in the outcome measures?
3. Eligibility criteria:
a. Both primary and secondary GBM patients are included. What is the justification for this? Secondary GBM are likely cancer patients that have lived longer through a first cancer and may have different physical function at baseline than those newly diagnosed with a primary GBM. Randomization will render the distribution of secondary GBM patients fairly equal between the two groups but it still seems like a heterogenous population
b. What grade is being included? In Feasibility, it states that should participant accrual be more challenging than anticipated we will open recruitment to grade III gliomas. Are only grade I and II included then?
4. The sample size calculation lacked supporting information. A proposed meaningful change of 0.28 was the basis for determining sample size. However, what is the standard deviation expected for functional performance in this population?

INVESTIGATORS:
This is an excellent team with complementary expertise in exercise oncology, exercise measurement, exercise physiology, behavioural medicine, medical oncology, exercise and clinical trials. There is a marked commitment to EDI in the composition of the team members but also in working with diverse populations within Nova Scotia.

ENVIRONMENT:
The environment is an established network of investigators that all target exercise in high risk populations like cancer survivors. There is clinical expertise on the team that can help in knowledge transfer.

RELEVANCE:
Given my concerns on the lack of preliminary data to support the feasibility of this trial, if GBM patients are unable to adhere to the exercise plan while undergoing treatment, then the impact of this study on patients living with GBM is low.

Budget:

Almost a quarter of the grant ($47,500) is budgeted for whole body magnetic resonance imaging which is the secondary outcome to capture body composition and overall, I felt that the secondary outcomes were lacking in their justification (probably due to space limitations).
