# Supplementary material for: The Impact of Resistance Exercise on Muscle Mass in Glioblastoma in Survivors (RESIST): Protocol for a Randomized Controlled Trial
Source: JMIR Res Protoc. 2022 May 4;11(5):e37709. doi: 10.2196/37709 (PMC9118089; doi:10.2196/37709)
Supplement: Multimedia Appendix 2 [file resprot_v11i5e37709_app2.pdf]

Applicant: Keats, Melanie

Institution: Dalhousie University

Application: The impact of Resistance ExerciSe on muscle mass in GIioblaSToma survivors (RESIST)

Review Team: Health Services/Social, Cultural, Environmental and Population Health

Review Report: Secondary Reviewer Report

Critique (consider research strategy, investigator(s), environment, and relevance):

Research Strategy:

This is a very well written proposal including good rationale for the proposed study, clear objectives, and well documented study design (clear inclusion/exclusion criteria, exercise intervention well described, outcome measures are well described and some are validated). However, the statistical analyses section could include more details (for example, how will patient age (and many other relevant co-variates) be adjusted for since it appears that age will not be accounted for in the randomization).

In an effort to increase sample size (currently proposed n=38) could recruitment of patients be done over a longer time period? (or in collaboration with an additional hospital?). It seems a shame not to be able to stratify the dataset by male/female (and the number of covariates may be larger than the sample size!). In addition, it is not clear why lower grade gliomas (and not just the highest grade, glioblastoma) are not included in this study.

Patients undergoing treatment and diagnosed with both primary and secondary glioblastoma will be recruited. However, would it be more appropriate to restrict this initial study to either primary or secondary? (assuming there are differences between these as regards patient symptoms and physical well being)

How will randomization be operationalized?

Investigators:
Dr. Melanie Keats (PI) is a Professor in the School of Health and Human Performance at Dalhousie University. She completed a PhD in 2007 (Exercise Psychology, University of Calgary). Her area of expertise is physical activity and cancer survivorship. Dr. Keats is a very active researcher and has been awarded 7 grants as PI/CoPI over the past 5 years. She also has a good publication record (23 papers over past 5 years).

Dr. Scott Grandy (Co-PI) is an Associate Professor in the School of Health and Human Performance at Dalhousie University. He completed a PhD in Pharmacology at Dalhousie University in 2005. Dr. Grandy has experience (as PI/CoPI) conducting several grant funded clinical exercise interventions to both prevent and treat cancer. He also has a good publication record.

Dr. Mary MacNeil (Co-PI) is a Medical Oncologist and Assistant Professor in the Medical Oncology Dept at the Queen Elizabeth Health Science Centre. She has extensive practical experience with the conduct of randomized trials among brain cancer patients. In addition to her research, Dr. MacNeil currently treats brain cancer patients.

The Co-investigators are all productive researchers: Dr. Chris Blanchard (Chair, Dept Medicine, Dalhousie University); Dr. Heather Neyedli (Associate Professor, Kinesiology Division, Dalhousie University); Dr Adrienne Weeks (Assistant Professor, Division of Neurosurgery, Dalhousie University).

An excellent team has been assembled which has the necessary experience and expertise to carry out the proposed project successfully.

Environment:
This interdisciplinary team of investigators has the necessary experience and expertise to carry out the proposed project successfully.

Relevance:
Patients undergoing treatment for glioblastoma (the most common malignant brain tumor) experience debilitating physical side-effects and toxicities which substantially reduce their quality of life. Physical exercise has been shown to be helpful in managing similar side-effects seen among patients with the more common cancers; however, few studies have explored the benefit of exercise specifically for glioblastoma patients. The proposed study is extremely relevant to improving the quality of life of patients suffering from glioblastoma.

Budget:

Seems appropriate.
