# Supplementary material for: The Impact of Resistance Exercise on Muscle Mass in Glioblastoma in Survivors (RESIST): Protocol for a Randomized Controlled Trial
Source: JMIR Res Protoc. 2022 May 4;11(5):e37709. doi: 10.2196/37709 (PMC9118089; doi:10.2196/37709)
Supplement: Multimedia Appendix 3 [file resprot_v11i5e37709_app3.pdf]

Applicant: Keats, Melanie

Institution: Dalhousie University

Application: The impact of Resistance ExerciSe on muscle mass in GIIoblaSToma survivors (RESIST)

Review Team: Health Services/Social, Cultural, Environmental and Population Health

Review Report: Tertiary Reviewer Report

Critique (brief comments focusing on the main strengths and weaknesses of the proposed research):

Main strengths: GBM represents an understudied cancer population. Interventions to improve quality of life are needed. Exercise is an attractive low-cost, low-risk complementary therapy in the treatment of cancer. The research team is experienced in exercise interventions studies.

Main weaknesses: Accrual and retention in exercise intervention studies can be difficult even in relatively well cancer populations. Patients targeted for this study will have undergone primary debulking surgery and be planned for RT + chemotherapy. Many will be overwhelmed by the diagnosis, prognosis, symptom burden, side effects and schedule of therapy, potentially limiting their ability and/or willingness to commit to a relatively intensive exercise intervention (3-4 sessions per week for 12 weeks). The multiple outcome measurements and surveys might also prove onerous for participants. The investigators' plan to mitigate this is to widen the study to grade III gliomas, if necessary. Additionally, study attrition might be quite high, though reasons for attrition will be explored.
